# Scaffold Functions of 14-3-3 Adaptors in B Cell Immunoglobulin Class Switch DNA Recombination

**DOI:** 10.1371/journal.pone.0080414

**Published:** 2013-11-25

**Authors:** Tonika Lam, Lisa M. Thomas, Clayton A. White, Guideng Li, Egest J. Pone, Zhenming Xu, Paolo Casali

**Affiliations:** 1 Institute for Immunology, School of Medicine and School of Biological Sciences, University of California Irvine, Irvine, California, United States of America; 2 Department of Microbiology and Immunology, School of Medicine, University of Texas Health Science Center at San Antonio, San Antonio, Texas, United States of America; Michigan State University, United States of America

## Abstract

Class switch DNA recombination (CSR) of the immunoglobulin heavy chain (*IgH*) locus crucially diversifies antibody biological effector functions. CSR involves the induction of activation-induced cytidine deaminase (AID) expression and AID targeting to switch (S) regions by 14-3-3 adaptors. 14-3-3 adaptors specifically bind to 5′-AGCT-3′ repeats, which make up for the core of all *IgH* locus S regions. They selectively target the upstream and downstream S regions that are set to undergo S–S DNA recombination. We hypothesized that 14-3-3 adaptors function as scaffolds to stabilize CSR enzymatic elements on S regions. Here we demonstrate that all seven 14-3-3β, 14-3-3ε, 14-3-3γ, 14-3-3η, 14-3-3σ, 14-3-3τ and 14-3-3ζ adaptors directly interacted with AID, PKA-Cα (catalytic subunit) and PKA-RIα (regulatory inhibitory subunit) and uracil DNA glycosylase (Ung). 14-3-3 adaptors, however, did not interact with AID C-terminal truncation mutant AIDΔ(180–198) or AIDF193A and AIDL196A point-mutants (which have been shown not to bind to S region DNA and fail to mediate CSR). 14-3-3 adaptors colocalized with AID and replication protein A (RPA) in B cells undergoing CSR. 14-3-3 and AID binding to S region DNA was disrupted by viral protein R (Vpr), an accessory protein of human immunodeficiency virus type-1 (HIV-1), which inhibited CSR without altering AID expression or germline I_H_-C_H_ transcription. Accordingly, we demonstrated that 14-3-3 directly interact with Vpr, which in turn, also interact with AID, PKA-Cα and Ung. Altogether, our findings suggest that 14-3-3 adaptors play important scaffold functions and nucleate the assembly of multiple CSR factors on S regions. They also show that such assembly can be disrupted by a viral protein, thereby allowing us to hypothesize that small molecule compounds that specifically block 14-3-3 interactions with AID, PKA and/or Ung can be used to inhibit unwanted CSR.

## Introduction

Immunoglobulin (Ig) class switch DNA recombination (CSR) and somatic hypermutation (SHM) are central to the maturation of the antibody response for the effectiveness of vaccines and the generation of neutralizing antibodies to microbial pathogens (including bacteria and viruses) and tumoral cells as well as the maturation of the autoantibody response in systemic or organ-specific autoimmunity. CSR irreversibly substitutes the Ig heavy chain (*IgH*) constant (C_H_) region, for instance, Cμ for IgM – which is expressed in all naïve B cells – with a downstream C_H_ region, Cγ, Cε or Cα [1], [2], thereby giving rise to IgG, IgE or IgA antibodies without altering the structure of the antigen-binding site [3], [4]. Class-switched IgG, IgE and IgA antibodies display different tissue distributions and possess diverse biological effector functions. SHM inserts mainly single nucleotide point-mutations into the antibody recombined V(D)J segments at a high rate to provide the structural substrate for positive selection of high affinity mutants by foreign antigen [5], [6].

CSR (as well as SHM) occur in B lymphocytes activated in peripheral lymphoid organs. CSR critically requires germline I_H_-C_H_ transcription of the upstream, e.g., Igμ, and downstream Igγ, Igε or Igα subloci [4]. Germline I_H_-C_H_ transcription initiates at the intervening Iμ, Iγ, Iε or Iα promoter and elongates through the I_H_ exon, intronic switch (S) region, and the C_H_ exon cluster, giving rise to germline Iμ-Cμ, Iγ-Cγ, Iα-Cα or Iε-Cε transcripts. CSR also requires activation-induced cytidine deaminase (AID, encoded by *AICDA* in humans and *Aicda* in mice), which is expressed at high levels in activated B lymphocytes, including those in germinal centers [7]–[9]. AID is a member of the AID/APOBEC cytidine deaminase family; it deaminates deoxycytosines (dCs) in S region DNA, yielding deoxyuracils (dUs) [10], [11]. The processing of dUs by uracil DNA glycosylase (Ung) results in abasic sites, nicking of which by apurinic/apyridimic endonucleases (APEs) leads to generation of DNA double-strand breaks (DSBs) in the upstream (donor, e.g., Sμ) and downstream (acceptor) S regions that are obligatory intermediates of CSR [12]. CSR then proceeds through DSB resolution [13]; synapsis of the upstream and downstream DSBs occurs through excision of the intervening DNA from the chromosome to form a switch DNA circle and leads to S–S DNA junctions. Switch DNA circles are transiently transcribed, giving rise to circle Iγ-Cμ, Iε-Cμ or Iα-Cμ transcripts, which are hallmarks of ongoing CSR [14]. Post-recombined *IgH* DNA sequences are transcribed, giving rise to post-recombination Iμ-Cγ, Iμ-Cε or Iμ-Cα transcripts, and mature V_H_DJ_H_-Cγ, V_H_DJ_H_-Cε or V_H_DJ_H_-Cα transcripts, which encode IgG, IgE or IgA, respectively [4].

Triggering of CSR requires both “primary” and “secondary” CSR-inducing stimuli [4]. Primary CSR-inducing stimuli comprise a T-dependent stimulus, i.e., engagement of CD40 expressed on B cells by trimeric CD154 expressed on CD4^+^ T cells, or a T-independent stimuli, such as dual engagement of Toll-like receptors (TLRs) and B cell receptor (BCR). Such dual engagement is exemplified by *E. coli* lipopolysaccharides (LPS), which engages TLR4 and BCR through its monophosphoryl lipid A and polysaccharide moieties, respectively [4], [15], [16]. Secondary CSR-inducing stimuli consist of cytokines such as interleukin-4 (IL-4), transforming growth factor-β (TGF-β) and interferon-γ (IFN-γ), which selectively and specifically induce germline Iγ-Cγ and Iε-Cε (IL-4) or Iγ2b-Cγ2b and Iα-Cα (TGF-β) or Iγ2a-Cγ2a (IFN-γ, in mouse but not human) transcription. Primary stimuli induce B cells to proliferate and express AID and other CSR-related genes. They also enable secondary stimuli to direct CSR to specific immunoglobulin isotypes [17], [18].

For CSR to unfold, AID and the whole CSR machinery must be targeted to the S regions that are set to undergo recombination to introduce DSBs, the resolution of which leads to S–S DNA recombination – dysregulation of AID expression and targeting has been associated with chromosomal translocations, lymphomagenesis and autoimmunity [19]–[22]. In species that use CSR to diversify their antibodies, all S region “cores”, within which DSBs and S–S junctions preferentially segregate, contain high-density repeats of the 5′-AGCT-3′ motif [23], [24]. 14-3-3 adaptor proteins (seven homologous isoforms, 14-3-3β, 14-3-3ε, 14-3-3γ, 14-3-3η, 14-3-3σ, 14-3-3τ and 14-3-3ζ) [25], [26] specifically bind to 5′-AGCT-3′ repeats and are selectively recruited to the upstream and downstream S regions that are set to undergo S–S DNA recombination by the H3K9acS10ph combinatorial histone modification [17], [23]. Once docked onto S regions, 14-3-3- adaptors mediate the assembly of macromolecular complexes on S region DNA. This notion is indeed supported by our demonstration that 14-3-3 adaptors recruit/stabilize AID and protein kinase A (PKA), which phosphorylates AID at serine (Ser)38, on S regions [23], [27], [28] and that that all seven 14-3-3 isoforms directly interact with AID and PKA catalytic subunit (PKA-Cα) and that 14-3-3γ, 14-3-3σ and 14-3-3ζ enhance AID dC DNA deamination activity [23].

Here, we hypothesized that 14-3-3 adaptors function as structural scaffolds to stabilize multiple enzymatic elements on S regions in CSR. To test our hypothesis, we adapted the bimolecular fluorescence complementation (BiFC) assay to quantify the direct interaction between all seven isoforms of 14-3-3 adaptors with CSR factors, such as AID, PKA, RPA, Ung, or with AID mutants or Ung mutants that cannot mediate CSR. We also used (GST) pull-down assay to show that 14-3-3 interact with AID, PKA and Ung. Finally, we quantified the direct interaction between 14-3-3, AID, PKA or Ung and viral protein R (Vpr), an accessory protein of the human immunodeficiency virus type-1 (HIV-1) and utilized this naturally occurring viral protein to disrupt 14-3-3 and AID binding to S region DNA, which inhibited CSR. Thus, our findings suggest that 14-3-3 adaptors play important scaffold functions and nucleate the assembly of multiple CSR factors on S regions and that small molecule compounds that specifically disrupt their interactions can be used to inhibit unwanted CSR, such as CSR underlying the generation of IgG and IgA autoantibodies in autoimmunity and atopic IgE antibodies in allergies and asthma.

## Materials and Methods

### B Lymphocytes

Spontaneously switching human sIgμ^+^ sIgδ^+^4B6 B cells and inducible switching human sIgμ^+^ sIgδ^+^2E2 B cells were derived from the CSR- and SHM-inducible human monoclonal sIgμ^+^ sIgδ^+^ CL-01 B cell line [23], [29], [30]. Single B cell suspensions were prepared from murine spleens using a 70 µm cell strainer. B cells were suspended in RPMI-1640 medium (Invitrogen) supplemented with FBS (10% v/v, Thermo Scientific), penicillin-streptomycin and amphotericin B fungizone (1% v/v) and 50 µM β-mercaptoethanol (FBS-RPMI) [16].

### Class Switch DNA Recombination

To analyze CSR by surface Ig, sIgμ^+^ sIgδ^+^ murine B cells were cultured at 10^5^ cell/ml in FBS-RPMI in the presence of LPS deproteinized by chloroform extraction (3 µg/ml, from *E. coli*, serotype 055:B5, Sigma-Aldrich) plus recombinant mIL-4 (4 ng/ml, R&D Systems) for CSR from IgM to IgG1. B cells were harvested and stained using phycoerythrin (PE)-labeled anti–mouse B220 mAb (clone RA3-6B2, BD Biosciences), 7–aminoactinomycin D (7–AAD, Sigma-Aldrich) and allophycocyanin (APC)-labeled anti–mouse IgG1 mAb (clone X56, BD Biosciences) using FACSCalibur™ flow cytometer (BD Biosciences). Dead (7–AAD^+^) cells were excluded from analysis.

### Bimolecular Fluorescence Complementation (BiFC)

BiFC assays were performed as we described [23]. Briefly, EYFP was split into two complementary moieties: the N-terminal 154 amino acids (EYFP1–154) and the C-terminal 84 amino acids (EYFP155–238). EYFP1–154 was fused with Flag–tagged AID or AID mutants, PKA-Cα, PKA-RIα, RPA1, Ung or Ung mutants, or 14-3-3ζ; EYFP155–238 was fused with influenza hemagglutinin (HA)–tagged 14-3-3β, ε, γ, η, σ, τ or ζ, or Vpr. 5×10^5^ HeLa cells cultured in DMEM (Invitrogen) supplemented with FBS, were transfected with 1 µg of plasmid using Lipofectamine™ (Life Technologies). After 24 hours, cells were analyzed for cell viability (7–AAD^–^) and for EYPF intensity by FACSCalibur™ flow cytometer (BD Biosciences); after 36 hours, cells were imaged for cell viability (ProLong Gold Antifade Reagent with 4′,6′-diamidino-2-phenylindole, DAPI, Invitrogen) and for EYFP intensity by an Olympus FluoView 1000 confocal microscope.

### Chromatin Immunoprecipitation (ChIP)

ChIP assays were performed as we described [23]. B cells were treated with 1% (v/v) formaldehyde for 10 min at 25°C to crosslink chromatin before being washed with cold PBS containing protease inhibitors (Roche) and resuspended in lysis buffer (20 mM Tris-HCl, 200 mM NaCl, 2 mM EDTA, 0.1% w/v SDS and protease inhibitors, pH 8.0). Chromatin was sonicated to yield DNA fragments (about 200 to 600 bps), pre-cleared with protein A agarose beads (Pierce) and then incubated with rabbit anti–14-3-3γ Ab (catalog # 18647, IBL, Inc.) or mouse anti–AID mAb (catalog # 39-2500, Invitrogen) overnight at 4°C. Immune complexes were precipitated by Protein A agarose beads, washed and then eluted with elution buffer (50 mM Tris-HCl, 0.5% SDS, 200 mM NaCl, 100 µg/ml proteinase K, pH 8.0), followed by incubation at 65°C for 4 hours to reverse formaldehyde cross-links and digest proteins. DNA in the supernatant was purified using a QIAquick PCR purification kit (Qiagen). Recovered DNA was specified by PCR using the following oligonucleotide primers: Sμ, forward 5′-GCTAAACTGAGGTGATTACTCTGAGGTAAG-3′ and reverse 5′-GTTTAGCTTAGCGGCCCAGCTCATTCCAGT-3′; Sγ1, forward 5′-ATAAGTAGTAGTTGGGGATTC-3′ and reverse 5′-CTCAGCCTGGTACCTTATACA-3′. Data were normalized to input chromatin DNA and depicted as enrichment of each amplicon DNA relative to baseline value obtained using an irrelevant mAb.

### *Aicda* and *IgH* locus Germline I_H_-C_H_, circle Iγ1- Cμ and Post-recombination Iμ-C_H_ Transcripts

RNA was extracted from 2×10^6^ cells using the RNeasy Mini Kit (Qiagen) according to the manufacturer’s protocol. Levels of *Aicda* transcripts, germline Iμ-Cμ and Iγ1-Cγ1 transcripts, circle Iγ1- Cμ transcripts and post-recombination Iμ-Cγ1 transcripts were quantified by reverse transcription (RT) and real-time quantitative PCR (qPCR). First-strand cDNAs were synthesized from equal amounts of total RNAs (2 µg) using the SuperScript™ III First-Strand Synthesis System (Invitrogen). Real-time qPCR analysis was performed using the DNA Engine Opticon Real-Time PCR Detection System (Bio-Rad Laboratories) to measure SYBR-green (DyNAmo HS SYBR Green) incorporation with the following protocol: 50°C for 2 min, 95°C for 10 min, 40 cycles of 95°C for 10 sec, 60°C for 20 sec, 72°C for 30 sec, 80°C for 1 sec, and data acquisition at 80°C, and 72°C for 10 min. Melting curve analysis was performed from 72°−95°C and samples were incubated for another 5 min at 72°C. The ΔΔCt method was used to analyze levels of transcripts and data were normalized to levels of *Gapdh* transcripts. For qPCR, the following primers were used: *Aicda* transcripts, forward 5′-TGCTACGTGGTGAAGAGGAG -3′ and reverse 5′-TCCCAGTCTGAGATGTAGCG-3′; germline Iμ-Cμ transcripts, forward 5′-ACCTGGGAATGTATGGTTGTGGCTT-3′ and reverse 5′- TCTGAACCTTCAAGGATGCTCTTG -3′; germline Iγ1-Cγ1 transcripts, forward 5′- TCGAGAAGCCTGAGGAATGTG -3′ and reverse 5′-ATGGAGTTAGTTTGGGCAGCA-3′; circle Iγ1- Cμ transcripts, forward 5′-GGCCCTTCCAGATCTTTGAG-3′ and reverse 5′-ACCTGGGAATGTATGGTTGTGGCTT-3′; post-recombined Iμ-Cγ1 transcripts, forward 5′-ACCTGGGAATGTATGGTTGTGGCTT -3′ and reverse 5′-ATGGAGTTAGTTTGGGCAGCA-3′; *Gapdh* transcripts, forward 5′-TTCACCACCATGGAGAAGGC-3′ and reverse 5′-GGCATGGACTGTGGTCATGA-3′.

### GST pull-down Assays

Spleen B cells (10^7^) were stimulated with LPS plus mIL-4 for 48 hours. Cells were collected and resuspended in lysis buffer (20 mM of Tris-Cl, pH 7.5, 150 mM of NaCl, 0.5 mM of EDTA, 0.25% (v/v) of NP-40) supplemented with phosphatase inhibitors Na_4_P_2_O_7_ (1 mM), NaF (10 mM) and NaVO_3_ (1 mM) and a cocktail of protease inhibitors (Sigma). After sonication and centrifugation, protein lysates were precleared with immobilized Glutathione Magnetic Beads (Pierce) bound to glutathione S-transferase (GST) for 1 hour at 4°C. Precleared whole cell lysates were then probed with immobilized Glutathione Magnetic Beads bound to either GST or GST–14-3-3γ for 1 hour at 4°C. After three times of washing with lysis buffer, protein complexes were eluted in the SDS sample buffer and fractionated by SDS-PAGE.

### Immunoblotting

Fractionated protein complexes were transferred onto polyvinylidene difluoride (PVDF) membranes (Thermo Scientific) at 4°C. After blocking and overnight incubation with rabbit anti–14-3-3γ Ab (catalog # 18647, IBL, Inc.), mouse anti–AID mAb (catalog # 39-2500, Invitrogen), rabbit anti–PKAα cat Ab (catalog # 903, Santa Cruz Biotechnology), rabbit anti–Ung Ab (catalog # 103236, GeneTex), mouse anti–GAPDH mAb (catalog # 239, GeneTex), mouse anti–Flag mAb (catalog # F3165, Sigma) or mouse anti–β-actin mAb (catalog # A5441, Sigma) the membranes were incubated with horseradish peroxidase (HRP)-conjugated secondary Abs. After washing with 0.05% PBS-Tween 20, bound HRP-conjugated Abs were detected using Amersham ECL Plus Western Blotting Detecting Reagents (GE Healthcare).

### Image Analysis

To detect codistributing molecules, human B cells were spun onto cover slips pre-coated with 10 µg/ml poly-D-lysine (Sigma-Aldrich), fixed with 2% paraformaldehyde and permeabilized with 0.25% Triton X-100. After blocking with 1% BSA, cells were stained with a rabbit anti–14-3-3 Ab (catalog # ab6081, Abcam) and a mouse anti–AID mAb (catalog # 39–2500, Invitrogen) or a mouse anti–RPA/p34 mAb (catalog # MS-691-P1, NeoMarkers), followed by staining with FITC-conjugated goat anti–rabbit and TRITC-conjugated goat anti–mouse IgG Ab. Confocal microscopy images of spontaneously switching 4B6 B cells cultured at 5×10^4^ cell/ml in (FBS-RPMI) and human 2E2 B cells stimulated with nil or anti–hCD40 mAb plus hIL-4 were captured by an LSM510-META NLO multi-photon confocal microscope and analyzed using the laser scanning microscope (LSM) Image Examiner software (Carl Zeiss Microimaging, Inc.). Each pixel in individual cells was analyzed for intensity in green (14-3-3) and red (AID or RPA). The ratio of the number of yellow pixels, to the total number of yellow and green pixels was used to quantify the percentage of 14-3-3 molecules codistributing with AID or RPA.

To detect nuclear foci, spontaneously switching human 4B6 B cells or primary mouse B cells stimulated with LPS plus mIL-4 were washed and then extracted in cytoskeletal (CSK) buffer to deplete soluble cytoplasmic and nuclear components before resuspended in 4% paraformaldehyde in PBS to crosslink chromosomal DNA and bound proteins. Cells were then processed, stained and immunofluorescence images were taken at multiple confocal planes. Such *z*-stacked images were de-convoluted to generate high-resolution images. All images were pseudo-colored for presentation. B cells were scored as positive for colocalization of 14-3-3 nuclear foci with AID nuclear foci or RPA nuclear foci when showing at least two colocalizing foci, defined as spots within the top 75% pixel intensity of each fluorescence on the pixel intensity map by the Autoquant® X software (Media Cybernetics, Inc.). The percentages of positive cells at 0 hour, 24 hours and 48 hours of stimulation were plotted against time points and the Mander's overlapping coefficiency were calculated to be between 0.91-0.99 (maximum is 1.0), thereby reflecting a time-dependent increase of colocalization. Processing and staining of mouse primary B cells or human 4B6 B cells were performed following a protocol for foci formation analysis [31].

### Retroviral Transduction

The coding sequence of GFP-Vpr (originally from the pGFP-Vpr plasmid, catalog # 11386, *The NIH AIDS Research and Reference Reagents Program*) was cloned into the retroviral vector pCSretTAC to generate pTAC-GFP-Vpr (generation of pTAC-GFP was described before [23]). Retroviral constructs were transfected along with the pCL–Eco retrovirus–packaging vector into HEK293T cells using the ProFection Mammalian Transfection System® (Promega). Transfected cells were cultured in FBS-RPMI in the presence of chloroquine (25 µM) for 8 hours. After the removal of chloroquine, retrovirus-containing culture supernatants were harvested every 12 hours for 48 hours. For transduction and CSR analysis, mouse B cells were activated with LPS for 24 hours and then centrifuged at 500 *g* together with viral particles in the presence of 6 µg/ml polybrene (Sigma-Aldrich) for 90 min at 25°C. Transduced B cells were then cultured in virus-free FBS-RPMI in the presence of LPS plus mIL-4 for 48 hours for transcript analysis, or for 72 hours for flow cytometry analysis of surface IgG1 and B220 expression in GFP^+^ B cells.

### HIV-1 Vpr (15-mer) Peptides

Twenty-two Vpr peptides, each of 15–amino acids in length and each with a sequential 11–amino acid overlap, covering the entire 96–amino acid viral protein, was provided by *The NIH AIDS Research and Reference Reagents Program* (catalog # 6447). Vpr peptides were dissolved in DMSO and then added to LPS-activated B cell cultures for 24 hours, after which B cells were stimulated with LPS plus mIL-4 for 72 hours to induce CSR from IgM to IgG1.

### Detection of Vpr in Human B Lymphocytes

Tonsils from HIV-1^–^ subjects and lymph nodes from HIV-1^+^ patients (UC Irvine Medical Center) were sectioned (7 µm) onto glass slides and stained with Abs specific to human using rabbit anti–Vpr Ab (catalog #11836, *NIH AIDS Research and Reference Reagents Program*) alone, or with mouse anti–human CD20 Ab (clone 2H7, eBioscience) or with mouse anti–AID mAb (catalog # 39–2500, Invitrogen). Color was development by DAB+ chromogen (Dako) and slides were dehydrated and counterstained with hematoxylin. Images were captured at 40X magnification using Nikon Eclipse E400.

To detect B cell uptake of Vpr, human 4B6 B cells were cocultured for 7 days with human CEM.NK^R^-CCR5 T cells infected with nil or HIV-192US657 (a clinical isolate of the primary R5 HIV-1 strain [32]–[34]), fixed with 2% paraformaldehyde and permeabilized with 0.25% Triton X-100 (Sigma-Aldrich). After blocking with 1% BSA, cells were stained with FITC-conjugated anti–μ mAb or FITC-conjugated anti–CD4 mAb to identify sIgμ^+^4B6 cells or CD4^+^ CEM.NK^R^-CCR5 T cells, respectively and rabbit anti–Vpr Ab (catalog # 11836, *NIH AIDS Research and Reference Reagents Program*) and Alexa 594-conjugated goat anti–rabbit IgG Ab to identify Vpr uptake for 1 hour. DAPI was used to stain the nucleus. Fluorescence images were captured at 100X magnification. All experiments involving HIV-192US657 were performed in a dedicated Biosafety Level III facility.

### Ethics Statement

Human materials. All human subject materials were surgical remnants collected and maintained for research purposes by the School of Medicine Department of Pathology, University of California, Irvine, CA 92697– under patient privacy rule, no information was disclosed on age, sex and identity of the patient sources of the surgical materials. The use of these materials for research associated with this study was specifically approved by the Institutional Review Board of the University of California, Irvine.

Mice. C57BL/6 mice 8–12 weeks old were housed in a pathogen-free facility and provided with autoclaved food and deionized water. The Institutional Animal Care and Use Committee of the University of California, Irvine, CA 92697, specifically approved the use of mice for the isolation of primary murine B cells for research associated with this study.

### Statistical Analysis

Statistic analysis was performed using Excel® software (Microsoft) to determine *P* values by two-tailed paired student’s *t*-test. *P* values less than 0.05 were considered significant.

## Results

### 14-3-3 Adaptors Bind AID, but not CSR-defective AID C-terminal Mutants

**Figure 1:**
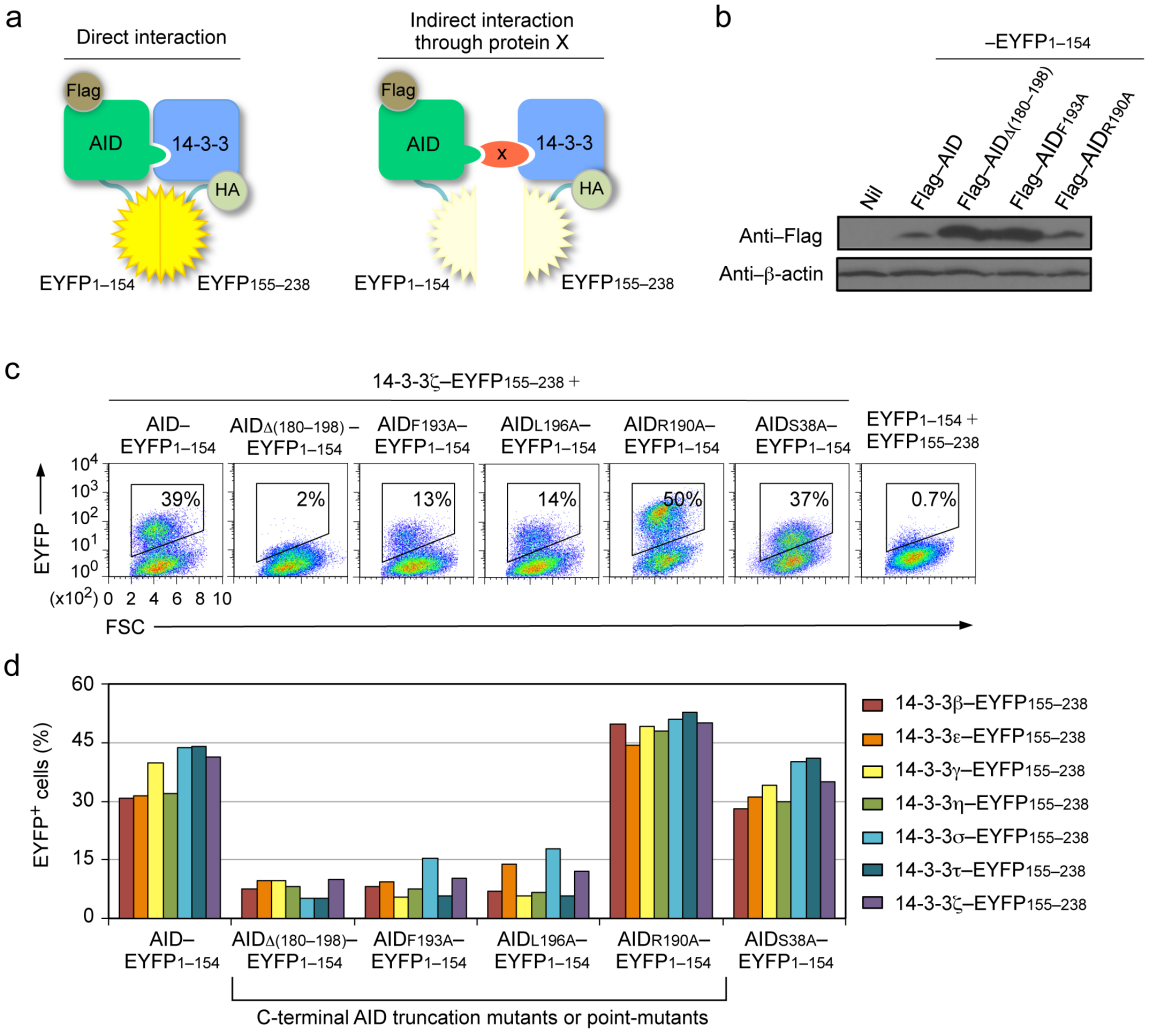
14-3-3 adaptors interact with AID through the AID C-terminus. (**a**) Schematics of the principle of the BiFC assays to analyze interaction of 14-3-3 (HA–14-3-3–EYFP155–238) and AID (Flag–AID–EYFP1–154). (**b**) Immunoblotting using specific mAbs to identify Flag and β-actin in HeLa cell expressing nil (pcDNA3 vector), Flag–AID, Flag–AIDΔ(180–198), Flag–AIDF193A, Flag–AIDR190A (fused to EYFP1–154). (**c**) BiFC assays of the interaction between 14-3-3ζ (fused to EYFP155–238) and AID, and AIDR190A and AIDS38A, but not AIDΔ(180–198), AIDF193A or AIDL196A (fused to EYFP1–154) in HeLa cells (at 24 hours), as analyzed by flow cytometry. (**d**) Quantification of the interaction between each of the seven 14-3-3 isoforms (β, ε, γ, η, σ, τ, ζ; fused to EYFP155–238) and AID, AIDΔ(180–198), AIDF193A, AIDL196A, AIDR190A or AIDS38A (fused to EYFP1–154), in HeLa cells (at 48 hours) depicted as percentage of EYFP^+^, as analyzed by flow cytometry. Data are representative of those from three independent experiments.

The direct interaction between 14-3-3 and AID was addressed using BiFC assays. In our BiFC assays, EYFP was split into two complementary moieties: the N-terminal 154 amino acids (EYFP1–154) and the C-terminal 84 amino acids (EYFP155–238) [35], [36]. EYFP1–154 was fused with Flag–tagged AID (Flag–AID–EYFP1–154), AID C-terminal truncation mutant (Flag–AIDΔ(180–198)–EYFP1–154), AID C-terminal point mutants (Flag–AIDR190A–EYFP1–154, Flag–AIDF193A–EYFP1–154, Flag–AIDL196A–EYFP1–154) or AID S38A mutant (Flag–AIDS38A–EYFP1–154). EYFP155–238 was fused with influenza hemagglutinin (HA)–tagged 14-3-3β, ε, γ, η, σ, τ or ζ, (HA–14-3-3β–EYFP155–238, HA–14-3-3ε–EYFP155–238, HA–14-3-3γ–EYFP155–238, HA–14-3-3η–EYFP155–238, HA–14-3-3σ–EYFP155–238, HA–14-3-3τ–EYFP155–238 or HA–14-3-3ζ–EYFP155–238, respectively). Upon coexpression of Flag–AID–EYFP1–154 and HA–14-3-3–EYFP155–238 in human HeLa cells, the two EYFP moieties complement each other and give off fluorescence complex (EYFP^+^) only when juxtaposed, as a result of direct interaction between AID and 14-3-3. By contrast, if AID and 14-3-3 interact though a third molecule, this ‘spacer’ molecule will prevent the direct interaction between AID and 14-3-3, and therefore, hamper EYFP1–154 and EYFP155–238 reciprocal complementation (**Figure 1a**). We have previously shown [23], and confirmed here, that all seven 14-3-3 isoforms interacted with AID and that 14-3-3ζ failed to interact with AIDΔ(190–198) or AIDΔ(180–198), which was expressed at a higher level than AID (**Figure 1b, 1c** and not shown), two AID C-terminal truncation mutants that are defective in mediating CSR despite their normal DNA dC deamination activity [37]. In addition, 14-3-3ζ displayed reduced (by 66%) interaction with AID C-terminal point-mutant AIDF193A (**Figure 1c**), which fails to mediate CSR, despite its predominant nuclear localization [38], [39]. 14-3-3ζ also displayed reduced interaction with another CSR-defective AID C-terminal point-mutant AIDL196A. By contrast, 14-3-3ζ directly interacted with AIDR190A (**Figure 1c**), which can fully rescue CSR in *Aicda* ^−*/*−^ B cells [38]. Like 14-3-3ζ, the other six 14-3-3 isoforms interacted with AIDR190A, but failed to interact with AIDΔ(180–198), AIDF193A or AIDL196A. Finally, all seven 14-3-3 adaptors directly interacted with AIDS38A mutant, which can be recruited to S region DNA [27], but cannot be phosphorylated by PKA and mediates limited CSR [28] (**Figure 1d**).

Thus, all seven isoforms of 14-3-3 adaptors interact with AID and a CSR-proficient AID C-terminal point-mutant, but not with CSR-defective AID C-terminal truncation mutant or point-mutants.

### 14-3-3 Adaptors bind PKA and Ung but not RPA

**Figure 2:**
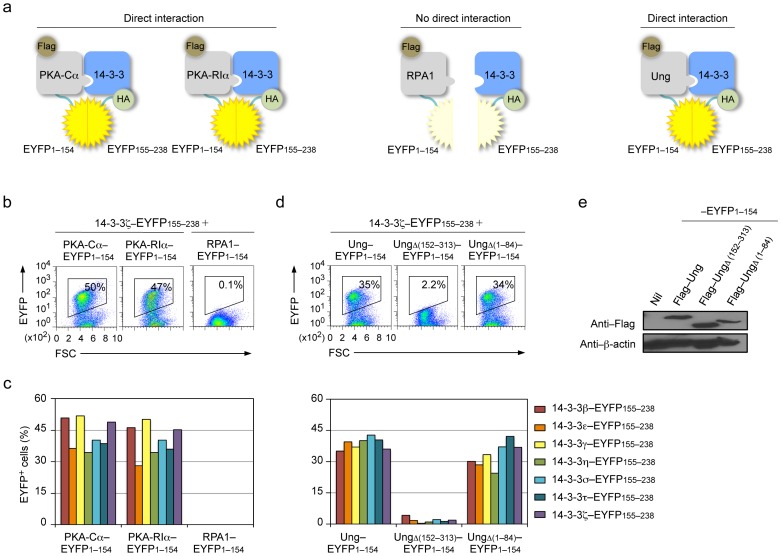
14-3-3 adaptors interact with PKA and Ung. (**a**) Schematics of the principle of the BiFC assays to analyze interaction of 14-3-3 (HA–14-3-3–EYFP155–238) with PKA-Cα (Flag–PKA-Cα–EYFP1–154), PKA-RIα (Flag–PKA-RIα–EYFP1–154), RPA1 (Flag–RPA1–EYFP1–154) or Ung (Flag–Ung–EYFP1–154). (**b**) BiFC assays of the interaction between 14-3-3ζ (fused to EYFP155–238) and PKA-Cα and PKA-RIα, but not RPA1 (fused to EYFP1–154) in HeLa cells, as analyzed by flow cytometry. (**c**) Quantification of the interaction between each of the seven 14-3-3 isoforms (β, ε, γ, η, σ, τ, ζ; fused to EYFP155–238) and PKA-Cα, and PKA-RIα or RPA1 (fused to EYFP1–154, left panel), and Ung, and UngΔ(152–313) or UngΔ(1–84) (fused to EYFP1–154, right panel) in HeLa cells depicted as percentage of EYFP^+^, as analyzed by flow cytometry. (**d**) BiFC assays of the interaction between 14-3-3ζ (fused to EYFP155–238) and Ung and N-terminal truncation mutant UngΔ(1–84), but not C-terminal truncation mutant UngΔ(152–313) (fused to EYFP1–154) in HeLa cells, as analyzed by flow cytometry. (**e**) Immunoblotting using specific mAbs to identify Flag and β-actin in HeLa cell expressing nil (pcDNA3 vector), Flag–Ung, Flag–UngΔ(152-313), Flag–UngΔ(1–84) (fused to EYFP1–154). Data are representative of those from three independent experiments.

The direct interaction between 14-3-3 and PKA-Cα and PKA-RIα or RPA1 was addressed using BiFC assays. The kinase activity of the two catalytic subunits PKA-Cα and PKA-Cβ within the PKA holoenzyme is inhibited by the two regulatory inhibitory subunits PKA-RIα and PKA-RIIα; upon allosteric binding of cAMP, these two regulatory subunits are released – PKA-RIα is the major PKA regulatory subunit and has been shown to interact with AID and be recruited to S region DNA, as part of the PKA holoenzyme [28], [40]. In our BiFC assays, HA–tagged 14-3-3β, ε, γ, η, σ, τ or ζ–EYFP155–238 and Flag–PKA-Cα–EYFP1–154, Flag–PKA-RIα–EYFP1–154 or Flag–RPA1–EYFP1–154 were coexpressed in human HeLa cells. All seven 14-3-3 adaptors directly interacted with PKA-Cα and PKA-RIα (**Figure 2a, 2b, 2c**). 14-3-3 adaptors, however, did not interact with RPA1 (**Figure 2a, 2b, 2c**), a 70 kDa subunit that binds single-stranded (ss) DNA [**41**].

The direct interaction between 14-3-3 and Ung was also addressed using BiFC assays. As we have previously shown, Ung is recruited/stabilized on S region DNA by Rev1, a translesion DNA synthesis (TLS) polymerase, which functions as a scaffold protein in CSR [42], through direct interactions to mediate CSR by processing dUs in S regions for the generation of high density of DSBs. In our BiFC assays, HA–tagged 14-3-3β, ε, γ, η, σ, τ or ζ–EYFP155–238 and Flag–Ung–EYFP1–154 and Ung N-terminal truncation mutant Flag–Ung Δ(1-84)–EYFP1–154 and Ung C-terminal truncation mutant Flag–UngΔ(152–313)–EYFP1–154 were coexpressed in human HeLa cells. All seven 14-3-3 adaptors directly interacted with Ung and with Ung N-terminal truncation mutant UngΔ(1-84), which can rescue CSR in *Ung* ^−*/*−^ B cells [43], [44]. 14-3-3 adaptors, however, did not bind Ung C-terminal truncation mutant UngΔ(152–313), which was expressed at a higher level than Ung or UngΔ(1-84) (**Figure 2c, 2d, 2e**).

Thus, all seven isoforms of 14-3-3 adaptors interact with both catalytic and regulatory subunits of PKA and with Ung in a manner dependent on Ung C-terminal amino acid residues, but not with RPA.

### 14-3-3γ Interacts with AID, PKA-Cα and Ung in B Cells Undergoing CSR

**Figure 3:**
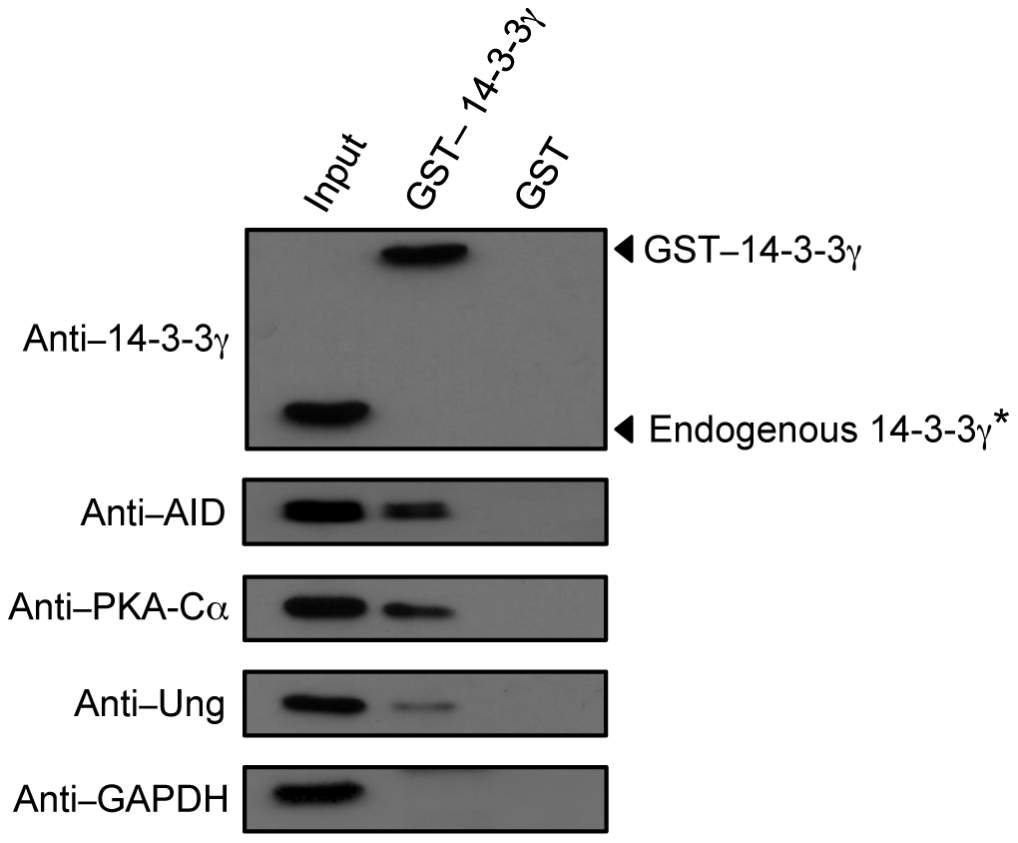
14-3-3γ interacts with AID, PKA-Cα and Ung expressed in B cells undergoing CSR. Immunoblotting using specific mAbs/Abs was performed to identify 14-3-3γ, AID, PKA-Cα and Ung in proteins pulled-down with GST–14-3-3γ or GST alone from whole lysates of mouse B cells stimulated with LPS plus mIL-4 for 48 hours. *Endogenous 14-3-3γ was detected when a higher amount of GST–14-3-3γ–precipitated proteins were analyzed, but no signal was detected for AID, PKA or Ung from GST–precipitated proteins. Data are representative of those from three independent experiments.

To confirm that 14-3-3γ can interact with AID, PKA-Cα and Ung in B cells undergoing CSR, we performed glutathione S-transferase (GST) pull-down experiments using 14-3-3γ fused to GST (GST–14-3-3γ) and whole cell lysates prepared from mouse B cells stimulated with LPS plus mIL-4 for 48 hours. Using immunoblotting, we readily detected AID, PKA-Cα and Ung among protein molecules after pull-down by GST–14-3-3γ, but not GST (**Figure 3**). GST-14-3-3γ was also detected, as expected, and endogenous 14-3-3γ was detectable when a higher amount of GST–14-3-3γ–precipitated proteins were analyzed (data not shown).

Thus, 14-3-3γ interacts with AID, PKA-Cα and Ung in switching B cells, consistent with its direct interactions with these CSR factors shown by our BiFC assays.

### 14-3-3 Adaptors Colocalize with AID and RPA in Nuclei of Switching B Cells

**Figure 4:**
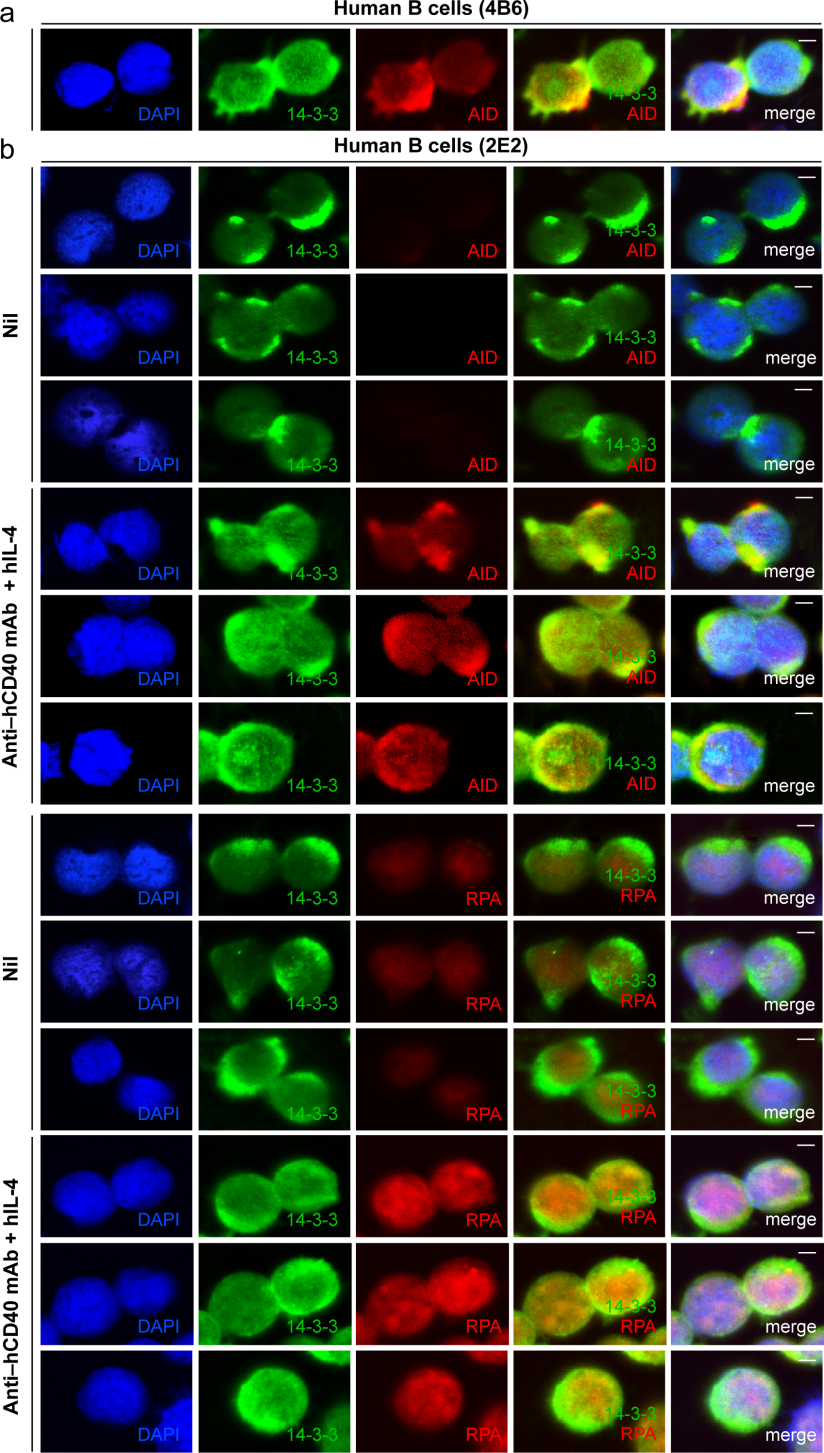
14-3-3 adaptors codistribute with AID and RPA in the nucleus of switching B cells. (**a**) Codistribution of 14-3-3 and AID in human 4B6 B cells (spontaneous CSR). (**b**) Codistribution of 14-3-3 with AID or RPA in human 2E2 B cells stimulated with agonistic anti–hCD40 mAb plus hIL-4 (to induce CSR to IgG1) for 48 hours. Scale bars: 5 μm. Data are representative of those from three independent experiments.

**Figure 5:**
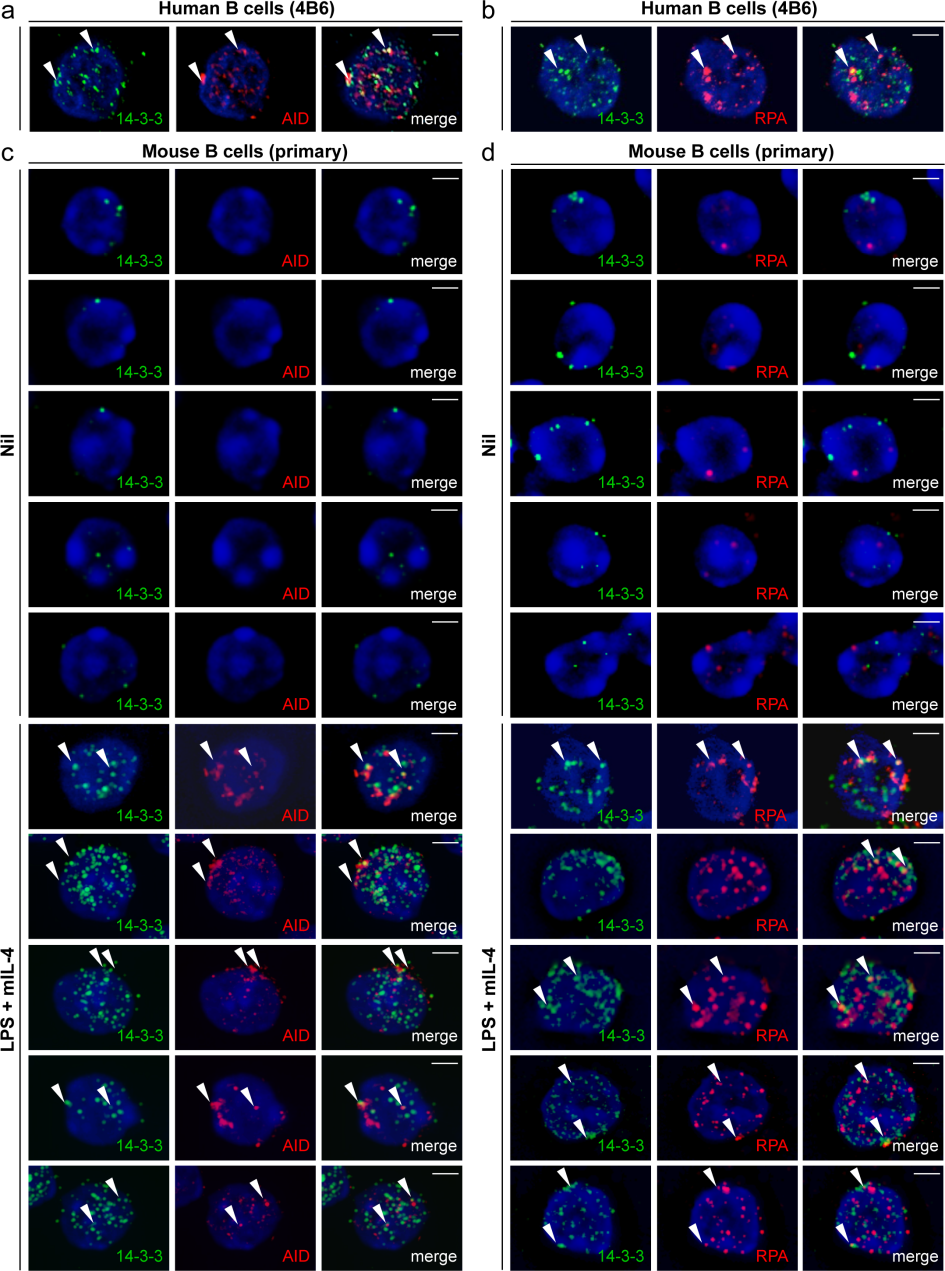
14-3-3 adaptors colocalize with AID and RPA in the nucleus of switching B cells. (**a**) Formation of 14-3-3 nuclear foci that colocalized with AID nuclear foci in human 4B6 B cells. (**b**) Formation of 14-3-3 nuclear foci that colocalized with RPA nuclear foci in human 4B6 B cells. (**c**) Formation of 14-3-3 nuclear foci that colocalized with AID nuclear foci in mouse primary B cells stimulated with LPS plus mIL-4 (to induce CSR to IgG1) for 48 hours. (**d**) Formation of 14-3-3 nuclear foci that colocalized with RPA nuclear foci in mouse primary B cells stimulated with LPS plus mIL-4 for 48 hours. Scale bars: 5 μm. Data are representative of those from three independent experiments.

**Figure 6:**
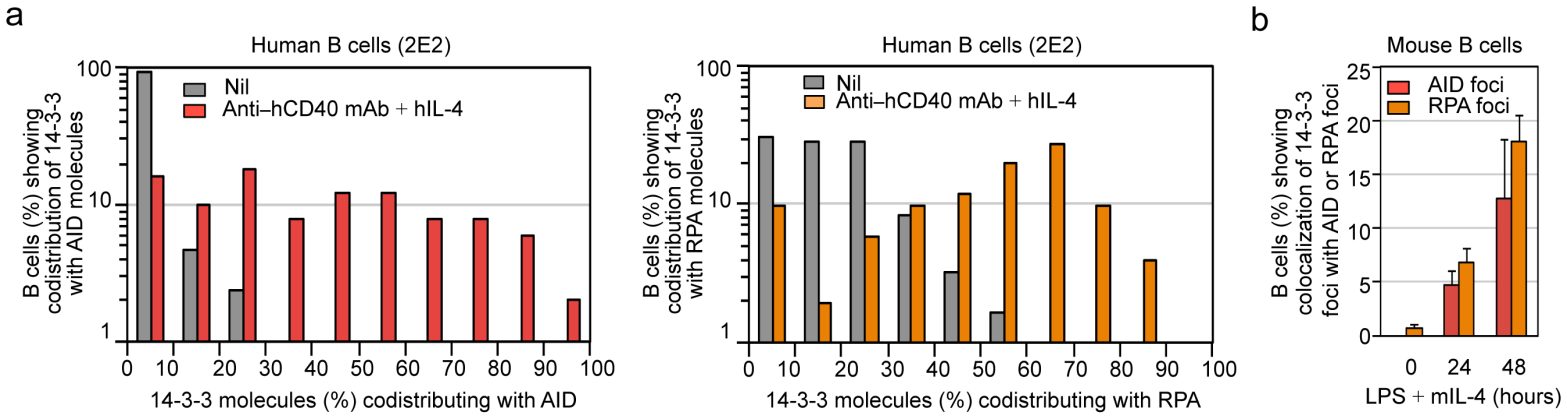
14-3-3 adaptors colocalize with AID and RPA at 24 hours and 48 hours. (**a**) Percentages of human 2E2 B cells showing 14-3-3 molecules codistributing (%) with AID (red) or RPA (orange) were calculated and depicted on the *y*-axis. At least 100 cells stimulated with nil or agonistic anti–hCD40 mAb plus hIL-4 were analyzed in each experiment. (**b**) Quantification of cells showing colocalization of 14-3-3 nuclear foci with AID nuclear foci or RPA nuclear foci in mouse primary B cells stimulated with LPS plus mIL-4 for 0 hour, 24 hours and 48 hours (mean and s.e.m. of data from three independent experiments).

As we have shown, 14-3-3ζ–AID interaction mainly occurs in the nucleus despite their predominant cytoplasmic localization [23]. Using confocal microscopy we found that, consistent with their direct interaction, 14-3-3 adaptors and AID were expressed and codistributed mainly in the cytoplasm and, to a lower degree, in the nucleus of human 4B6 B cells, which switch spontaneously from IgM to IgG, IgA and IgE [23], [45] (**Figure 4a**). Also, 14-3-3 codistributed with AID and RPA in human 2E2 B cells upon stimulation by an agonistic anti–hCD40 mAb plus hIL-4, which induce human 2E2 B cells to undergo CSR from IgM to IgG and IgE [23] (**Figure 4b**). To directly visualize the colocalization of 14-3-3 and AID or RPA in the nucleus, where CSR occurs, we adapted a protocol to deplete human and mouse B cells of cytosolic constituents and then identified 14-3-3, AID or RPA by specific Abs. As detected by this approach, 14-3-3, AID and RPA formed nuclear foci in spontaneously switching human 4B6 B cells (**Figure 5a, 5b**) and in mouse B cells stimulated by LPS plus mIL-4 to undergo CSR from IgM to IgG1 at high levels, after 24 and 48 hours (**Figure 5c, 5d**). We consistently detected two to four 14-3-3–containing nuclear foci that colocalized with AID–containing nuclear foci or RPA–containing nuclear foci (**Figure 5**), in spite of no direct interaction between 14-3-3 and RPA (**Figure 2b**). The percentages of human 2E2 B cells showing 14-3-3 molecules codistributing (%) with AID or RPA that were stimulated with agonistic anti–hCD40 mAb plus hIL-4 were higher than those of 2E2 B cells stimulated with nil (**Figure 6a**). The proportion of B cells that displayed colocalization of 14-3-3 nuclear foci with AID nuclear foci or RPA foci increased over time of stimulation from 0% to 4.7% within 24 hours, to 12.7% within 48 hours for AID, or from 0.8% to 6.8% within 24 hours, to 18.1% within 48 hours for RPA, consistent with increased proportions of IgG1^+^ B cells (typically 5% within 48 hours, 15% within 72 hours and 30% within 96 hours, not shown) under the same stimulation conditions (**Figure 6b**).

Thus, 14-3-3 adaptors colocalize with AID and RPA in the nucleus of human and mouse B cells undergoing CSR.

### 14-3-3 and AID Recruitment to S Regions and B Cell CSR are Inhibited by Vpr

**Figure 7:**
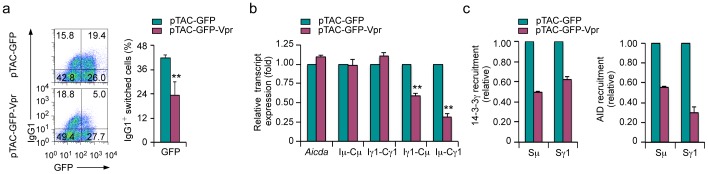
Inhibition of CSR and disruption of 14-3-3 recruitment to S regions by Vpr. (**a**) Proportions of IgG1^+^ cells in mouse primary B cells transduced with pTAC-GFP-Vpr or pTAC-GFP retrovirus (which mediated expression of GFP-Vpr or GFP, respectively) and stimulated with LPS plus mIL-4 for 72 hours (left; to induce CSR to IgG1), and quantification of class-switched surface IgG1^+^ B cells expressing GFP (right; mean and s.e.m of data from three independent experiments), as analyzed by flow cytometry. (**b**) Levels of *Aicda*, germline Iμ-Cμ and Iγ1-Cγ1, circle Iγ1-Cμ and mature post-recombination Iμ-Cγ1 transcripts in mouse primary B cells expressing GFP-Vpr or GFP and stimulated with LPS plus mIL-4 for 48 hours. Data were normalized to the level of *Gapdh* transcripts and depicted as the ratio of expression of transcripts in B cells expressing GFP-Vpr to that in B cells expressing GFP (mean and s.e.m of data from three independent experiments). **, *P*<0.01, t-test. (**c**) ChIP-qPCR analysis of the binding of 14-3-3γ and AID to the Sμ and Sγ1 region DNA in mouse primary B cells expressing GFP-Vpr or GFP and stimulated with LPS plus mIL-4 for 48 hours. Data were normalized to input chromatin DNA and depicted as enrichment of each DNA amplicon relative to baseline value obtained using irrelevant Ab. Data are representative of those from three independent experiments.

Prompted by our findings that 14-3-3 adaptors directly interacted and colocalized with AID and other CSR factors in the nucleus of switching B cells, we hypothesized that 14-3-3–mediated recruitment of the AID-centered CSR machinery to S regions would be blocked when interactions of 14-3-3 with CSR factors were disrupted by naturally occurring or synthetic molecules, leading to CSR inhibition. To test this hypothesis, we first analyzed CSR inhibition by utilizing the HIV-1 accessory protein Vpr, which has been suggested to bind to 14-3-3 proteins [46], Ung [43] and possibly PKA [47]. Primary mouse B cells were transduced with pTAC retrovirus to express green fluorescent protein (GFP) alone (pTAC-GFP) or Vpr linked to GFP (pTAC-GFP-Vpr) and then stimulated with LPS and mIL-4 for CSR from IgM to IgG1. CSR was inhibited by Vpr, as shown by reduced proportions of IgG1^+^ cells (23%) among B cells expressing GFP-Vpr when compared to their counterparts (42%) expressing GFP (**Figure 7**).

**Figure 8:**
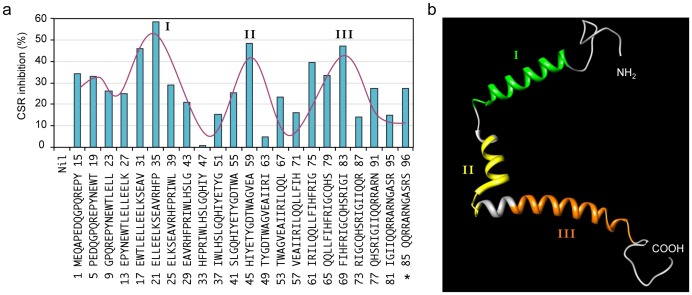
Vpr peptides inhibit CSR through segregated inhibitory clusters. (**a**) Percent of inhibition of CSR to IgG1 in mouse primary B cells stimulated with LPS plus mIL-4 in the presence of 22 sequential Vpr peptides (*denotes the C-terminal peptide, which is 12–amino acid long). Three Vpr peptide clusters with the highest CSR inhibitory effects are depicted as I, II and III. Data are representative of those from three independent experiments. (**b**) The three Vpr peptide clusters with the highest CSR inhibitory effects correspond to the three α-helices of Vpr. The graph was rendered by the UCSF Chimera^®^ software using the NMR structure of Vpr (PDB ID 1esx).

In addition, levels of circle Iγ1-Cμ and post-recombination Iμ-Cγ1 transcripts, which are accurate parameters of ongoing and completed CSR, respectively, were reduced in the presence of Vpr. However, levels of AID expression and germline Iμ-Cμ and Iγ-Cγ1 transcription were normal in B cells expressing Vpr (**Figure 7b**). To further dissect CSR inhibition by Vpr, we used an array of 22 Vpr peptides, each of 15– amino acid in length and each with a sequential 11–amino acid overlap, covering the entire 96–amino acid viral protein. We analyzed CSR inhibition by each peptide in mouse B cells induced by LPS plus mIL-4– uptake of these peptides by B cells was efficient, possibly due to their small size. A differential CSR inhibitory activity was displayed from different Vpr peptides (without affecting B cell proliferation or survival, data not shown), which segregated into three inhibitory clusters (I, II and III) (**Figure 8a**). The three clusters correlated with the three α-helices of Vpr [48], which are structurally independent (**Figure 8b**), and perhaps serve as distinct functional domains for the interaction of the retrovirus with host cell proteins.

**Figure 9:**
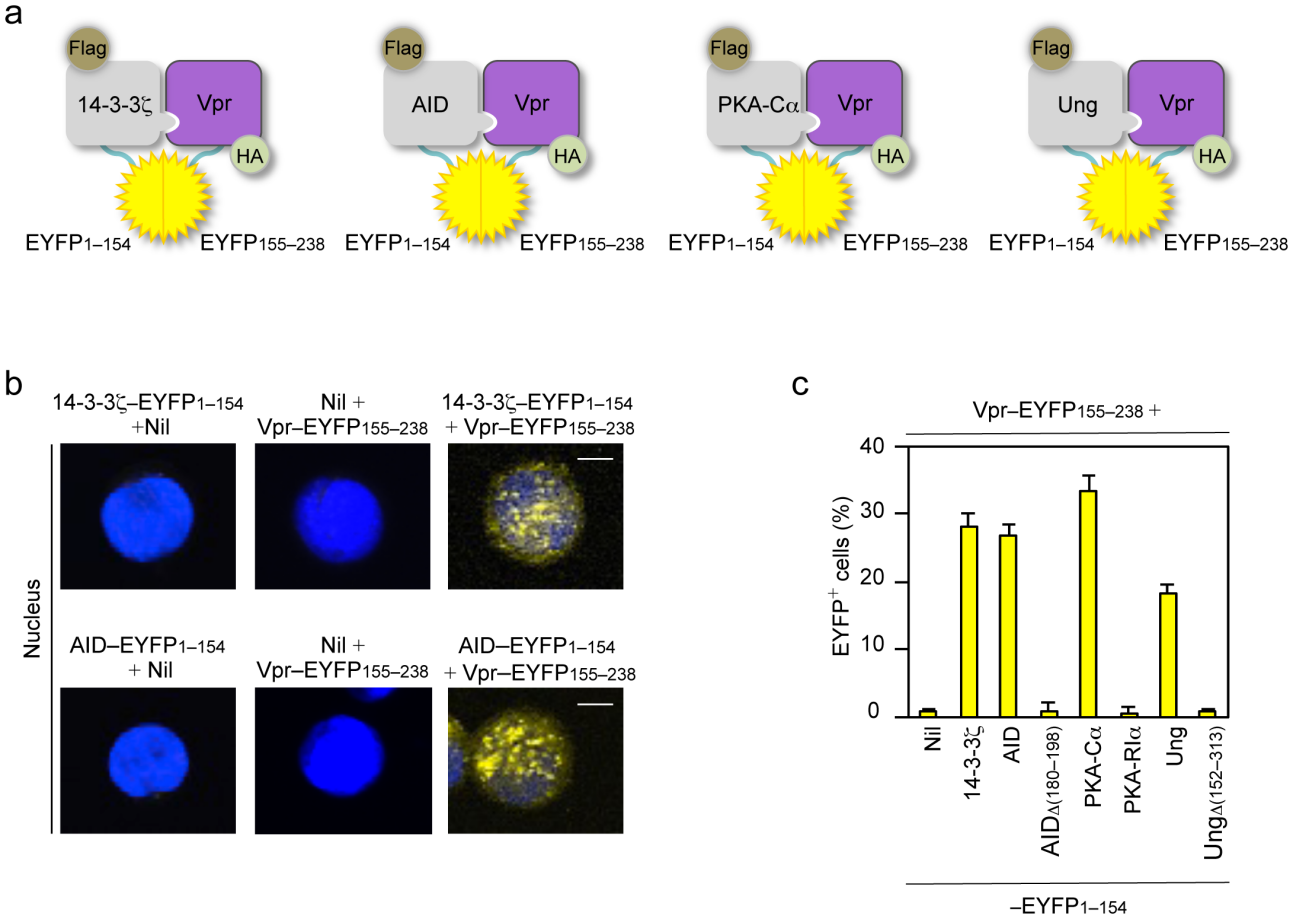
Essential CSR factors interact with Vpr. (**a**) Schematics of the principle of the BiFC assays to analyze interaction of 14-3-3ζ (Flag–14-3-3ζ–EYFP1–154), AID (Flag–AID–EYFP1–154), PKA-Cα (Flag–PKA-Cα–EYFP1–154) or Ung (Flag–Ung–EYFP1–154) with Vpr (HA–Vpr–EYFP155–238). (**b**) BiFC assays of the interaction between 14-3-3ζ or AID (fused to EYFP1–154) and Vpr (fused to EYFP155–238) in HeLa cells. DAPI (blue) was used to visualize the nucleus. Scale bars: 5 μm. (**c**) Quantification of the interaction between nil (pcDNA3 vector), 14-3-3ζ, AID, AIDΔ(180–198), PKA-Cα, PKA-RIα, Ung or UngΔ(152–313) and Vpr in HeLa cells depicted as percentage of EYFP^+^, as analyzed by flow cytometry. Data are representative of those from three independent experiments.

To address the molecular mechanisms underlying CSR inhibition by Vpr, we analyzed 14-3-3γ and AID recruitment to S regions in the presence of Vpr. Binding of 14-3-3γ and AID to Sμ and Sγ1 DNA was inhibited by Vpr, as shown by our ChIP assays using a specific Ab to 14-3-3γ or AID and chromatin from B cells expressing GFP-Vpr or GFP that were stimulated with LPS plus mIL-4 (**Figure 7c**). Further, BiFC assays were utilized to address the direct interaction between 14-3-3 or AID and Vpr by coexpressing Flag–14-3-3ζ–EYFP1–154 or Flag–AID–EYFP1–154 and HA–Vpr–EYFP155–238, respectively, in HeLa cells. 14-3-3ζ and AID indeed interacted with Vpr and they did so in the nucleus (**Figure 9a, 9b**). Further, in our BiFC assays involving Flag–AIDΔ(180–198)–EYFP1–154, Flag–PKA-Cα–EYFP1–154, Flag–PKA-RIα–EYFP1–154, Flag–Ung–EYFP1–154 or Flag–UngΔ(152–313)–EYFP1–154 with HA–Vpr–EYFP155–238, we showed that PKA-Cα and Ung, but not AIDΔ(180–198), PKA-RIα or UngΔ(152–313) interacted with Vpr (**Figure 9c**).

Thus, 14-3-3 adaptors function as scaffolds in mediating the recruitment of AID and possibly PKA and Ung to S regions, which can be inhibited by Vpr, likely due to the disruption of interactions between 14-3-3 and with those CSR factors, leading to the inhibition of CSR.

### AID Colocalize with Vpr in Germinal Center B Cells from HIV-1 Patients

**Figure 10:**
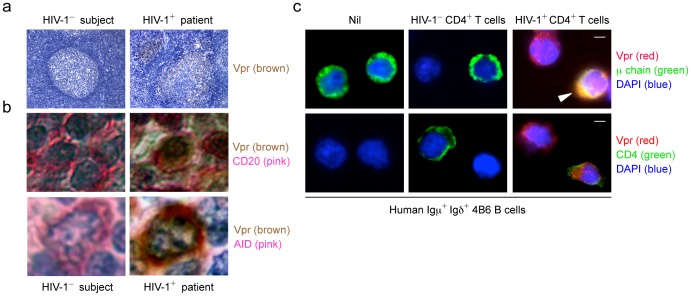
AID colocalized with Vpr in germinal center B cells from HIV-1^+^ patients. (**a**) Immunohistochemistry analysis of Vpr in germinal centers in the tonsil from an HIV-1^–^ subject (left) or in lymph nodes from an HIV-1^+^ patient (right). Original magnification 100×. (**b**) Immunohistochemistry analysis of Vpr and CD20 (top) or Vpr and AID (bottom) in the tonsil from an HIV-1^–^ subject (left) or in lymph nodes from an HIV-1^+^ patient (right). Original magnification 100X. (**c**) Immunofluorescence staining and confocal microscopy analysis of Vpr in human 4B6 B cells cocultured with nil (left), CEM.NK^R^-CCR5 T cells (middle) or CEM.NK^R^-CCR5 T cells infected with HIV-192US657 (right). Human 4B6 B cells and CEM.NK^R^-CCR5 T cells were stained by FITC-conjugated anti–μ Ab (top) and anti-CD4 mAb (bottom), respectively. DAPI (blue) was used to visualize the nucleus. Scale bars: 5 μm. Data are representative of those from three independent experiments.

We next addressed the biological relevance of CSR inhibition by Vpr during HIV infection, in which a decrease in class-switched antibodies has been reported to occur [49]. HIV does not infect B cells, but the HIV component such as Nef have been detected in B cells [49]. This prompted us to hypothesize that Vpr is present in secondary lymphoid structures during HIV infection. Here, we analyzed lymph node sections from HIV-1^+^ patients and tonsil sections from HIV-1^–^ subjects by immunohistochemistry using a specific Ab to Vpr. This revealed Vpr localized within germinal centers of HIV-1^+^ patients but not HIV-1^–^ subjects (**Figure 10a**). In addition, AID colocalized with Vpr within germinal center B cells of lymph nodes from HIV-1^+^ patients but not HIV-1^–^ subjects after double staining with an anti–Vpr Ab and anti–AID Ab or anti–CD20 Ab (**Figure 10b** **)**.

To demonstrate that B cells can acquire Vpr from an HIV-1^+^ source, we cocultured (HIV-1^–^) human 4B6 B cells with (HIV-1^–^) human CEM.NK^R^-CCR5 T cells infected with nil or HIV-192US657 (a clinical isolate of the primary R5 HIV-1 strain [32]-[34]). 4B6 B cells displayed Vpr nuclear and cytoplasmic internalization, as indicated by the intracellular staining with FITC-conjugated anti–μ chain mAb staining and with Alexa 594-conjugated anti–Vpr Ab, only when cocultured with HIV-1-infected T cells, which were specified by staining with FITC-conjugated anti–CD4 mAb and positive for the intracellular staining of Vpr (**Figure 10c**).

Thus, AID colocalizes with Vpr within germinal center B cells of HIV-1^+^ patients and can be picked up by HIV-1^–^ B cells upon coculturing with HIV-1^+^ T cells.

## Discussion

The recruitment and scaffold functions of 14-3-3 adaptors for the stabilization of enzymatic activity of AID and other CSR factors on S regions require multiple molecular interactions. Here, we showed that all seven isoforms of 14-3-3 adaptors directly interacted with AID, but failed to interact with AID C-terminal point mutants that have been shown to be defective in mediating CSR [38], [39]. In B cells undergoing CSR, 14-3-3 and AID formed nuclear foci of macromolecular complexes, as suggested by the colocalization of 14-3-3 nuclear foci and AID nuclear foci at 24 hours and 48 hours, but not at 0 hour. Non-colocalizing 14-3-3 nuclear foci may be involved in binding to H3K9acS10ph in the genome (outside the *IgH* locus) for transcription regulation [50]; non-colocalizing AID foci may be responsible for dC deamination outside the Ig locus [51]-[53], particularly in regions where RNA polymerase II stalls [54], [55].

All seven isoforms of 14-3-3 adaptors directly interacted with the catalytic and regulatory subunits of the PKA holoenzyme. These would lead to 14-3-3–mediated stabilization of CSR factors on S region DNA and enhancement of AID and PKA activity. 14-3-3 adaptors also directly interacted with Ung, which together with APEs mediates the dominant pathway of DSB generation in CSR [4], [12]. The direct interaction of 14-3-3 adaptors with Ung likely serves two important functions. First, 14-3-3 adaptors possibly facilitate AID and Ung reciprocal stabilization on S region DNA [56], as 14-3-3 possibly bridge AID and Ung to form an AID–14-3-3–Ung complex. This notion is further supported by findings that the reciprocal and cooperative stabilization of AID and Ung depends on the AID C-terminal region, which, as we showed [23], mediates 14-3-3–AID interaction. Second, 14-3-3 may enhance the processing of AID-inserted dUs by Ung for the eventual generation of DSBs. This putative 14-3-3 function would complement and/or enhance that of Rev1, which serves as a scaffold for Ung by stabilizing Ung on S region DNA and enhancing its enzymatic activity [42]. Mismatch repair (MMR) proteins Msh2 and Msh6, recognize deoxyuracil:deoxyguanine mismatches and recruit 5′→3′ exonuclease I (ExoI) to the generate DSBs [57], and they stabilize AID on S region DNA in a manner dependent on AID C-terminal region [56]. 14-3-3 adaptors have been shown to interact with ExoI [58], [59], suggesting that 14-3-3 adaptors are also involved in MMR-dependent DSB generation. The critical importance of the interaction between 14-3-3 adaptors and essential CSR factors, likely contribute to the assembly of the CSR machinery, which includes AID, Ung, Msh2 and Msh6 [56], [57].

14-3-3 nuclear foci colocalized with RPA nuclear foci despite no direct interaction between 14-3-3 and RPA1, the ssDNA-binding subunit, suggesting that RPA1 can only interact with 14-3-3 through another CSR-related factor, such as phosphorylated AID at S38 to form an RPA–AID–14-3-3 complex. RPA has been suggested to mark AID-mediated DNA damage sites [60] and can bind several CSR factors involved in DSB generation, such as Ung [61], MMR elements [62] and the Mre11/Rad50/Nbs1 complex [63]. RPA may also be key to the transition from formation of DSB-generating macromolecular complexes to DSB-resolving complexes on S regions, as RPA can also bind factors that are involved in CSR DSB resolution, such as γ-H2AX, 53BP1 and DNA-PKcs [4].

CSR to IgG1 in primary B cells was significantly reduced in the presence of HIV-1 Vpr; Vpr was also reported to inhibit CSR to IgA in CH12F3 B cells [43]. The CSR inhibitory activities of Vpr segregated within three structural clusters (I, II and III) corresponding to the three Vpr helices, therefore, our studies suggest the notion that CSR inhibition by Vpr is through a “multi-prong” strategy. Interestingly, cluster I peptides encompassed the Ung-binding Vpr region [64] and cluster III peptides straddled the 14-3-3- and PKA-interacting Vpr regions [47], [65]. Inhibition of CSR by Vpr did not affect AID expression or germline I_H_-C_H_ transcription, suggesting that unlike HIV-1 Nef, which inhibits CSR by interfering with CD40-signaling and NF-κB activation [66], signaling events induced by primary CSR-stimuli and/or secondary CSR-stimuli is not affected by Vpr. By contrast, intimate CSR mechanisms was likely disrupted by Vpr, particularly the binding of 14-3-3 to S region 5′-AGCT-3′ repeats, to H3K9acS10ph, or to important CSR factors, such as AID, PKA and Ung. This notion is supported by our data that recruitment of 14-3-3γ to Sμ and Sγ1 DNA was disrupted by Vpr, and that essential CSR factors such as 14-3-3ζ, AID, PKA-Cα and Ung directly interacted with Vpr. Reduced binding of 14-3-3γ to S regions, perhaps in conjunction with putative blocking of 14-3-3 and AID interaction by Vpr, lead to reduced AID recruitment to Sμ and Sγ1 DNA. Importantly, proliferation, survival or cell cycle of B cells was not affected in the presence of Vpr, by contrast Vpr–mediated cycle arrest in T cells has been reported [67].

As we have also shown here, AID colocalized with Vpr in HIV-1^+^ patients lymph node germinal center B cells. In addition, HIV-1^–^ B cells internalized Vpr when cocultured with HIV-1-infected CD4^+^ T cells. B cell internalization of HIV-1 Vpr is likely facilitated by CD21 [68], [69], CD40 and/or binding of DC-SIGN [70] – Vpr can be released from virions or infected T cells in an extracellular form in an extracellular form [71], which can be internalized by bystander B cells through protein transduction [72]. Irrespective of Vpr entry route, CSR and the generation of class-switched IgG, possibly including HIV neutralizing IgG antibodies [49], [66], is efficiently inhibited by Vpr, leading to HIV-1 evading immune response and persistently infecting the host.

**Figure 11:**
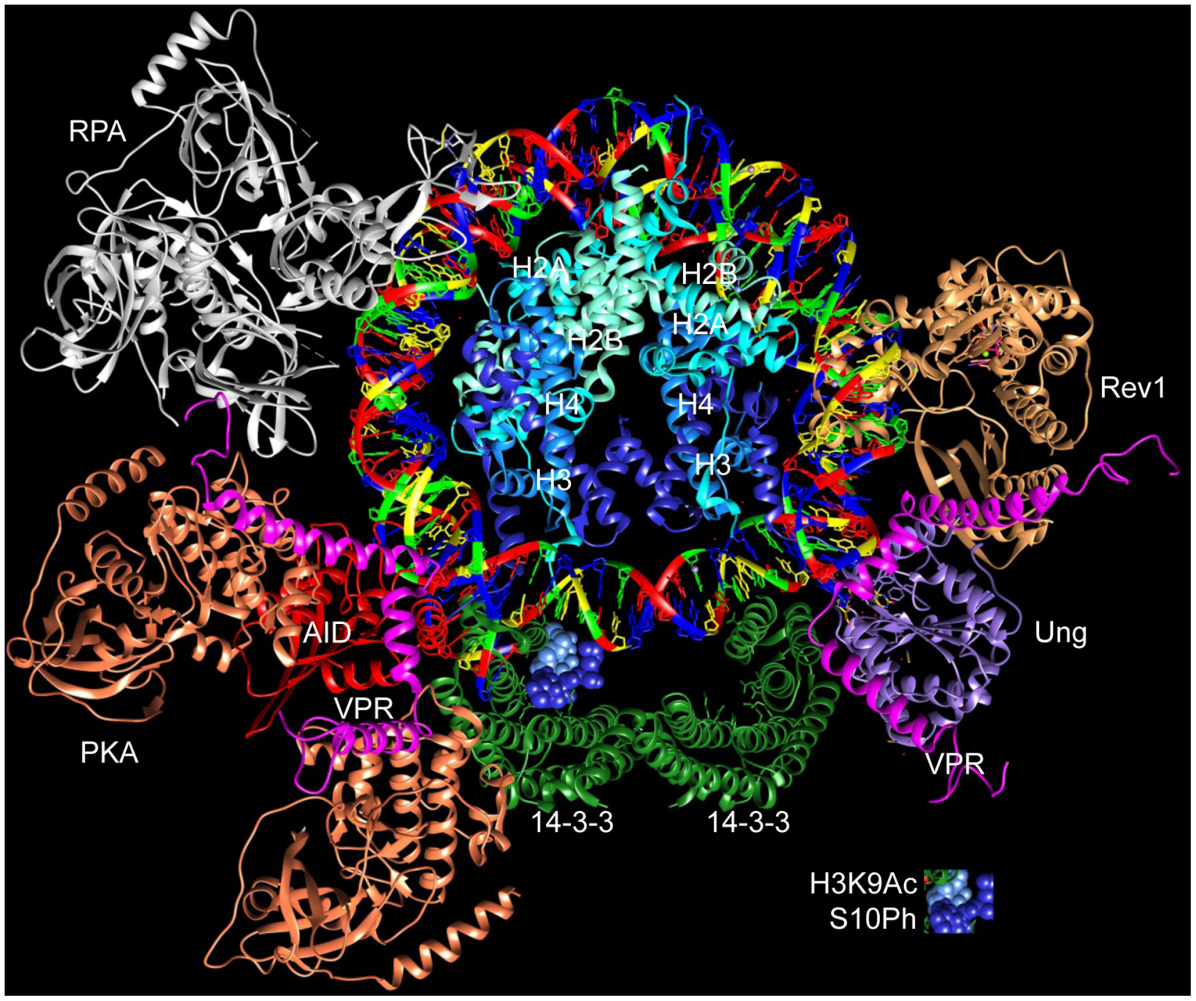
Schematic illustration of 14-3-3 scaffold functions in the assembly of S region DNA-protein complexes and disruption of such complexes by the naturally occurring Vpr molecule. A speculative model of a possible configuration of interacting proteins during CSR, and the disruption of some of these complexes by Vpr, as suggested by our current and previous findings [4], [23], [42]. The CSR macromolecular complex localizes to chromatin S regions through 14-3-3 adaptors (green) binding to the modified histone tail H3K9acS10ph (PDB ID 2c1j) as well as to 5′-AGCT-3′ repeats (PDB ID 3afa), which specifically recur in S region DNA. Upon recruitment to S regions by 14-3-3, AID (red, homology modeled after human APOBEC2, PDB ID 2nyt) is phosphorylated at Ser38 by the PKA catalytic subunit (orange, PDB ID 3tnp), leading to the recruitment of RPA (silver, PDB ID 4gop) to S region DNA. Rev1 (brown, PDB ID 3gqc) is important for CSR by virtue of its ability to stabilize Ung (purple, PDB ID 3fci) on S region DNA. Vpr (magenta, PDB ID 1esx) impairs CSR, possibly by disrupting critical DNA–protein interactions, such as S region DNA–14-3-3 binding, and/or protein–protein interactions, such as those between 14-3-3 and AID, PKA or Ung. Graphics were rendered using the UCSF Chimera software (http://www.cgl.ucsf.edu/chimera).

Overall, our findings provide evidence that 14-3-3 adaptor proteins nucleate the assembly of the AID-centered CSR machinery on 5′-AGCT-3′ DNA repeats and H3K9acS10ph marks in S regions that are set to undergo S–S recombination (**Figure 11**). They also suggest that CSR DNA–protein and/or protein–protein complexes can be destabilized by naturally occurring molecules, leading to inhibition of CSR, thereby providing a basis for the identification of synthetic small molecule compounds, such as those that disrupt DNA–protein and/or protein–protein interactions involving 14-3-3 [26], [73], or biologics that can effectively inhibit unwanted CSR, such as CSR underlying the generation of IgG and IgA autoantibodies in autoimmunity and atopic IgE antibodies in allergies and asthma.
