# Correction: Scaffold Functions of 14-3-3 Adaptors in B Cell Immunoglobulin Class Switch DNA Recombination

**DOI:** 10.1371/journal.pone.0174195

**Published:** 2017-03-15

**Authors:** Tonika Lam, Lisa M. Thomas, Clayton A. White, Guideng Li, Egest J. Pone, Zhenming Xu, Paolo Casali

There is an error in Fig 2D. The flow cytometry plot showing the interaction of 14-3-3ζ with UngΔ (1–84) (right panel) in the BiFC assay was an erroneous duplication of the plot showing the interaction of 14-3-3ζ with Ung (left panel). The authors have provided a correct flow cytometry plot for Fig 2D here.

**Fig 2:**
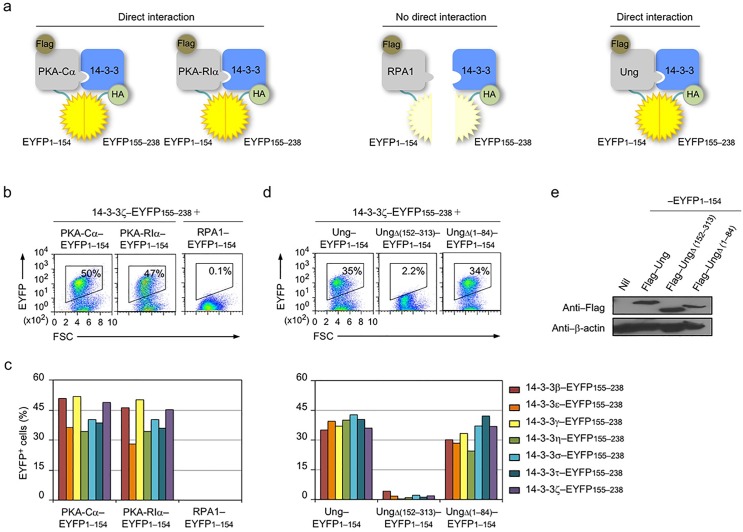
14-3-3 adaptors interact with PKA and Ung. (a) Schematics of the principle of the BiFC assays to analyze interaction of 14-3-3 (HA–14-3-3–EYFP155–238) with PKA-Cα (Flag–PKA-Cα–EYFP1–154), PKA-RIα (Flag–PKA-RIα–EYFP1–154), RPA1 (Flag–RPA1–EYFP1–154) or Ung (Flag–Ung–EYFP1–154). (b) BiFC assays of the interaction between 14-3-3ζ (fused to EYFP155–238) and PKA-Cα and PKA-RIα, but not RPA1 (fused to EYFP1–154) in HeLa cells, as analyzed by flow cytometry. (c) Quantification of the interaction between each of the seven 14-3-3 isoforms (β, ε, γ, η, σ, τ, ζ; fused to EYFP155–238) and PKA-Cα, and PKA-RIα or RPA1 (fused to EYFP1–154, left panel), and Ung, and UngΔ(152–313) or UngΔ(1–84) (fused to EYFP1–154, right panel) in HeLa cells depicted as percentage of EYFP^+^, as analyzed by flow cytometry. (d) BiFC assays of the interaction between 14-3-3ζ (fused to EYFP155–238) and Ung and N-terminal truncation mutant UngΔ(1–84), but not C-terminal truncation mutant UngΔ(152–313) (fused to EYFP1–154) in HeLa cells, as analyzed by flow cytometry. (e) Immunoblotting using specific mAbs to identify Flag and β-actin in HeLa cell expressing nil (pcDNA3 vector), Flag–Ung, Flag–UngΔ(152–313), Flag–UngΔ(1–84) (fused to EYFP1–154). Data are representative of those from three independent experiments.

## References

[1] Lam T, Thomas LM, White CA, Li G, Pone EJ, Xu Z, et al (2013) Scaffold Functions of 14-3-3 Adaptors in B Cell Immunoglobulin Class Switch DNA Recombination. PLoS ONE 8(11): e80414 doi:10.1371/journal.pone.0080414 24282540 10.1371/journal.pone.0080414 PMC3840166

